# Interhemispheric synchrony in the neonatal EEG revisited: activation synchrony index as a promising classifier

**DOI:** 10.3389/fnhum.2014.01030

**Published:** 2014-12-23

**Authors:** Ninah Koolen, Anneleen Dereymaeker, Okko Räsänen, Katrien Jansen, Jan Vervisch, Vladimir Matic, Maarten De Vos, Sabine Van Huffel, Gunnar Naulaers, Sampsa Vanhatalo

**Affiliations:** ^1^Division STADIUS, Department of Electrical Engineering (ESAT), University of LeuvenLeuven, Belgium; ^2^iMinds-KU Leuven Medical IT DepartmentLeuven, Belgium; ^3^Department of Development and Regeneration, University of LeuvenLeuven, Belgium; ^4^Department of Signal Processing and Acoustics, Aalto UniversityEspoo, Finland; ^5^Department of Psychology, University of OldenburgOldenburg, Germany; ^6^Department of Engineering Science, Institute of Biomedical Engineering, University of OxfordOxford, UK; ^7^Department of Children's Clinical Neurophysiology, HUS Medical Imaging Center and Children's Hospital, Helsinki University Central Hospital and University of HelsinkiHelsinki, Finland

**Keywords:** interhemispheric synchrony, biomarker, preterm infant, brain monitoring, neonatal EEG

## Abstract

A key feature of normal neonatal EEG at term age is interhemispheric synchrony (IHS), which refers to the temporal co-incidence of bursting across hemispheres during trace alternant EEG activity. The assessment of IHS in both clinical and scientific work relies on visual, qualitative EEG assessment without clearly quantifiable definitions. A quantitative measure, activation synchrony index (ASI), was recently shown to perform well as compared to visual assessments. The present study was set out to test whether IHS is stable enough for clinical use, and whether it could be an objective feature of EEG normality. We analyzed 31 neonatal EEG recordings that had been clinically classified as normal (*n* = 14) or abnormal (*n* = 17) using holistic, conventional visual criteria including amplitude, focal differences, qualitative synchrony, and focal abnormalities. We selected 20-min epochs of discontinuous background pattern. ASI values were computed separately for different channel pair combinations and window lengths to define them for the optimal ASI intraindividual stability. Finally, ROC curves were computed to find trade-offs related to compromised data lengths, a common challenge in neonatal EEG studies. Using the average of four consecutive 2.5-min epochs in the centro-occipital bipolar derivations gave ASI estimates that very accurately distinguished babies clinically classified as normal vs. abnormal. It was even possible to draw a cut-off limit (ASI~3.6) which correctly classified the EEGs in 97% of all cases. Finally, we showed that compromising the length of EEG segments from 20 to 5 min leads to increased variability in ASI-based classification. Our findings support the prior literature that IHS is an important feature of normal neonatal brain function. We show that ASI may provide diagnostic value even at individual level, which strongly supports its use in prospective clinical studies on neonatal EEG as well as in the feature set of upcoming EEG classifiers.

## Introduction

Recent progress in basic neuroscience, neuroimaging, and neonatal care has raised interest in the understanding of physiological and pathological processes in the preterm and neonatal brain. Electroencephalography (EEG) is a non-invasive and sensitive tool for evaluating brain function in the neonatal period. A key component in early brain functional development is the emergence of functional networks, the long range connectivity between and within brain hemispheres, which lay the basis for the development of neurocognitive capabilities (Uhlhaas et al., 2010; Lubsen et al., 2011; Omidvarnia et al., 2014). In this context, it is intriguing that synchrony between hemispheres has been considered as an important feature of normal neonatal brain function since the seminal studies in the late 1970s (Lombroso, 1979).

Clinical classification of spontaneous neonatal EEG is traditionally based on visual assessment of multiple features: continuity, quality of sleep wake organization, interhemispheric synchrony (IHS), symmetry and amplitude. However, visual EEG interpretation requires expertise, and there are no objective standards for classification schemes, nor any other established method to yield appropriate diagnostic accuracy to support modern neuroscience or evidence based medicine. This has greatly compromised comparisons of results between different studies. A quantitative analysis of EEG activity, including automated analysis of selected features of cortical function, could create an objective and appropriate classification scheme for neonatal EEG. Moreover, it could also support and accelerate visual EEG analyses in medical centers without access to highly skilled EEG interpretation.

Visual estimation of IHS is based on observation of co-incidence of bursts in spontaneous background EEG activity during tracé alternant/discontinue (Holmes and Lombroso, 1993). IHS is interpreted as a sign of connectivity or functional interaction between hemispheres and is hence considered an important feature of normal brain function, resulting from the development of callosal connections (Kostovic and Jovanov-Milosevic, 2006; Dudink et al., 2008).

Despite the long and widespread clinical use of IHS, there is no physiologically plausible definition of IHS for visual EEG reading (for more details, please see Räsänen et al., 2013). A quantitative measure for IHS, the activation synchrony index (ASI), was recently developed by Räsänen et al. (2013). The ASI is based on statistically measuring the temporal delay between two signal energies, and was shown to correlate with visually rated IHS grades. It was also shown to clearly outperform other methods proposed for the same purpose in the recent literature (Räsänen et al., 2013).

Our ultimate aim is to use ASI more widely as a clinical biomarker or as a feature in EEG classifiers, which poses special requirements for individual stability. In addition, the analysis settings such as the epoch lengths need to be practical with respect to clinical reality, where obtaining longer high quality epochs is often challenging due to intermittent, trivial artifacts. The aim of this study was to test the ability of the ASI to distinguish normal and abnormal neonatal EEG recordings. Therefore, we first defined the optimal ASI parameters to maximize its intraindividual and technical stability, and then tested ASI as an input for a classifier to identify abnormal EEG activity.

## Materials and methods

**Figure 1:**
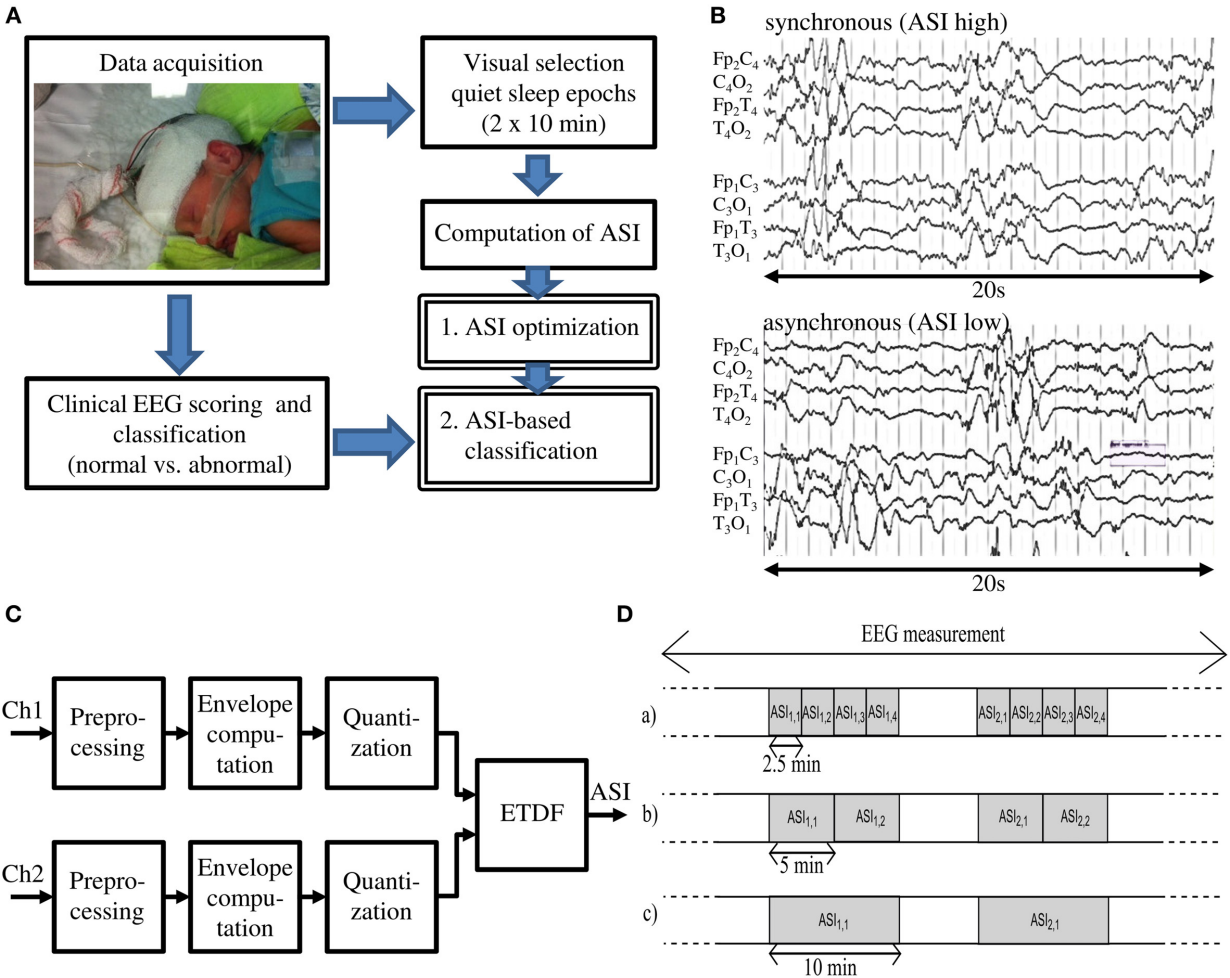
**(A)** Schematic overview of the presented method. **(B)** EEG tracks with Normal and Abnormal synchrony. **(C)** Schematic view of the ASI algorithm (Räsänen et al., 2013). **(D)** Two 10-min quiet sleep epochs are subdivided into shorter epochs of 2.5, 5, and 10 min for analysis, which gives respectively *ASI2.5, ASI5*, and *ASI10* values.

The present study consisted of two discrete phases (see Figure 1). First, we optimized the intraindividual stability of ASI. The earlier development of ASI had aimed to optimize parameter settings for maximal distinction between asynchrony grades as well as robustness against artifacts. Our present study continued ASI optimization by searching for EEG epoch lengths and channel derivations that would strike a balance between technical reliability and clinical practice. For clinical practice, an important consideration is that the algorithm should work equally well on EEG samples either with longer continuous epochs or with multiple shorter epochs. In addition, we tested our optimized ASI protocol on a new EEG dataset that had been classified using conventional clinical criteria, with the aim of seeing how well ASI alone is able to distinguish normal and abnormal EEG recordings. This latter aim is also a rigorous test of the conventional, albeit quantitatively untested assumption, that interhemispheric asynchrony would be an essential property in the abnormal EEG near term age.

### Subjects and EEG recordings

A total of 31 neonatal EEG traces [postmenstrual age (PMA) 36–42 weeks], were retrospectively selected by an expert (A.D.) to assess the spontaneous background EEG activity. These EEG recordings were collected from a larger EEG dataset, recorded for clinical purposes. All EEG data were recorded at 256 Hz with a video-synchronized EEG device (BRAIN RT, OSG equipment, Mechelen, Belgium). After skin preparation (Nuprep Gel), 10–17 Ag/AgCl cup electrodes were placed according to the international 10–20 standard locations. The reference electrode was Cz. Electrode impedance was below 10 kΩ at the start, and the signal quality was monitored visually throughout the recording. The minimum recording time was 4 h to record multiple vigilance states. The protocol was reviewed and approved by the relevant Ethics Committee of the University Hospitals of Leuven, Belgium.

The EEG records were reviewed for clinical purposes by two independent raters (A.D. and K.J.) who were not aware of the later use of the EEG for this study. Hence, they were fully blinded to both the overall design as well as the numerical ASI results. The clinical assessment is based on holistic, conventional visual criteria (see Table 1 in Supplementary Material). If there was any difference in the interpretation of an EEG, a consensus was reached after re-evaluation (2 patients, Table 1 in Supplementary Material). The following parameters were assessed on the whole measurement, according to standard developmental features in preterm and term EEG (Scher et al., 2005; André et al., 2010; Shellhaas et al., 2011; Hayashi-Kurahashi et al., 2012): (1) brain activity cycling as being normal or abnormal for the age; (2) grade of continuity; (3) age-specific landmarks: amplitude, disorganization, and dysmaturation patterns; and (4) qualitative synchrony in quiet sleep. The overall clinical classification of the EEG recording as normal (*n* = 14) vs. abnormal (*n* = 17) was based on observing at least two features that were not appropriate for age.

### Activation synchrony index

The ASI algorithm implemented in Matlab is described in full detail in the original publication (Räsänen et al., 2013). It takes two EEG-signals (e.g., bipolar derivations in left and right hemispheres, respectively) as inputs to be processed through four main stages (Figure 1C): (1) preprocessing; (2) computation of signal amplitude envelope; (3) quantization of the amplitude envelope; and (4) calculation of the ASI value, a single scalar value, from the temporal relationship of the two quantized signals. Technical details of the algorithm can be found in the Supplementary Material. For as long as the peaks in energy envelopes are temporally co-incident (clinically perceived as “synchronized”), their dependency is highest at zero lag and diminishes with increasing relative lag between the two channels, thereby leading to a high ASI value. On the other hand, lack of temporal co-incidence, the signature of low-synchrony, will result in low ASI (for example signals, see Figure 1B or Räsänen et al., 2013).

### Parameter optimization

The prior work on ASI development defined the main parameter settings, the most important of which appeared to be the definition of the frequency band, and the weighting of higher frequencies (a.k.a. “pre-emphasis”). These yielded an ASI that can discriminate between different visually rated IHS grades, and which clearly outperforms the other methods proposed earlier in the literature (Räsänen et al., 2013). However, it was not shown (1) how stable the ASI is technically and physiologically within an individual; (2) what window length would allow the most practical setting in clinical practice where selecting longer data epochs is always challenged by the intermittent, trivial artifacts; and (3) which of the bipolar EEG derivations would produce the best results.

#### Stability over time

It is commonly assumed by clinicians that IHS is a fairly stable property of a given brain function, at least at the scale of multiple hours. Thus, the IHS measure should be comparable between successive sleep cycles, and at least between few-minute epochs within a given QS period. Theoretically and physiologically thinking, it is also possible, and may even be more likely, that subtle fluctuations occur in the IHS over time. The clinically required intraindividual stability of our measure by a test-retest paradigm is technically straightforward, but the differences to be seen in repeated measures are affected by multiple factors. First, there is a numerical uncertainty in the measure itself when recorded from typically noisy neonatal recordings. Second, the feature quantified by ASI may change due to subtle fluctuations in brain state at multi-minute scale. The former possibility is typically tackled by using longer data epochs, while the latter possibility is better assessed by looking at multiple shorter data epochs. Hence, we decided to study the whole range of epoch lengths and numbers that we felt could be practical in future clinical implementations.

To this end, we divided the original 10-min data epochs into 10, 5, and 2.5-min epochs, and examined ASI in each. The aim was to define the epoch length with the smallest possible intrapatient deviations. This was assessed by searching for the lowest mean-squared difference (MSD) between the (average) ASI value of the first 10-min epoch (*ASI*_1_) and the (average) ASI value of the second 10-min epoch (*ASI*_2_). In formula 1 this difference is squared, summed and averaged over all patients, with *n* representing the number of patients.

(1) $$MSD\;=\;\frac{1}{n}\sum_{i=1}^{n}{\left(ASI_{1,i}\;-\;ASI_{2,i}\right)}^{2}$$

MSD measures the difference between the different ASI values within an individual. The optimal ASI parameters were expected to give the lowest MSD. In this way, a MSD minimization problem is solved as a function of different channel pair combinations and different ASI window lengths.

A further issue to investigate was the stability of ASI between different quiet sleep epochs. In clinical practice, it may not always be possible to find long epochs of undisturbed and good signal quality epochs of quiet sleep. Therefore, more data could be readily gathered from successive sleep cycles. Hence, we analyzed either continuous 20-min epochs from one cycle or two 10-min epochs from successive cycles.

#### ASI window length

ASI is a statistical estimate of synchrony, and its numerical accuracy (“technical reliability”) will increase when the analysis epochs get longer. Our earlier work showed that an ASI estimate becomes more stable when the epochs grow longer than about 2 min (Räsänen et al., 2013). Longer EEG epochs are needed to statistically quantify temporal co-incidence of pseudoperiodic, intermittent cortical activity. In this work, we searched for the optimal window by calculating the ASI feature for window lengths of 2.5, 5, and 10 min. To have a fair comparison between different window lengths, we studied two manually-selected epochs of 10 min. First, we divided each epoch into 4 equal parts of 2.5 min from which we separately computed the ASI value (*ASI2.5*), and then averaged them (Figure 1D upper part). Similarly, the 10-min epoch was split into two equal parts of 5 min and the ASI values (*ASI5*) were estimated and averaged (Figure 1D middle part). These average ASI values were compared to the single ASI value from the 10-min analysis (*ASI10*) (Figure 1D lower part).

#### Channel pair combination

IHS is traditionally analyzed from bipolar derivations (Lombroso, 1979) rather than from individual EEG signals (Omidvarnia et al., 2014). To comply with this idea, we also analyzed ASI from symmetric bipolar derivations. We wanted to define the derivation with least intraindividual variability. Hence, we computed ASI from the following symmetric combinations; Fp_1_C_3_-Fp_2_C_4_, C_3_O_1_-C_4_O_2_, Fp_1_O_1_-Fp_2_O_2_, Fp_1_T_3_-Fp_2_T_4_, T_3_O_1_-T_4_O_2_.

### Classification

After optimization of the ASI parameters, we examined the ability of ASI to distinguish normal and abnormal EEG recordings classified by clinical criteria. To this end, we used 20 min of data, channel pair combination C_3_O_1_-C_4_O_2_, and 2.5-min ASI analysis windows *(ASI2.5)*. This gave us two average ASI estimates in each patient, derived from the two 10-min epochs (Figure 1D upper part). The lower of these was used to represent the lowest possible synchrony level in the given patient (*ASI_class_*).

(2) $$ASI_{class}\;=\;minimum\left(mean_{1}\;ASI2.5,\;mean_{2}\;ASI2.5\right)$$

Next, we searched for a threshold that could distinguish normal and abnormal EEG records. The optimal ASI settings were found to yield nearly non-overlapping distributions of ASI values in the two groups, so the ASI threshold can be readily drawn between the distributions. However, we then wanted to see how much ASI-based classification would be compromised if the user has only a limited amount of data available for the ASI computation. In this situation, distributions between ASI values in the two groups are overlapping. Hence, all thresholds will have their own classifier performance, i.e., levels sensitivity and specificity. Their dependence on the threshold can be readily visualized using receiver operating characteristic (ROC) curves that were computed to assess the classifier performance as a function of thresholds.

### Age dependence of ASI

Finally, we studied the dependence of ASI on PMA to know whether it should be taken as a confounder in babies near term age. It is known that both anatomical and electrical cortico-cortical connectivity increases toward term age (Kostovic and Judas, 2010; Omidvarnia et al., 2014). The previous study with ASI showed a modest age-dependence in a much younger group of preterm babies compared to our present group of fullterm newborns (Räsänen et al., 2013). This could be a biological confounder when using ASI on babies with a wider age range.

## Results

### Optimal parameters for ASI stabilization

**Figure 2:**
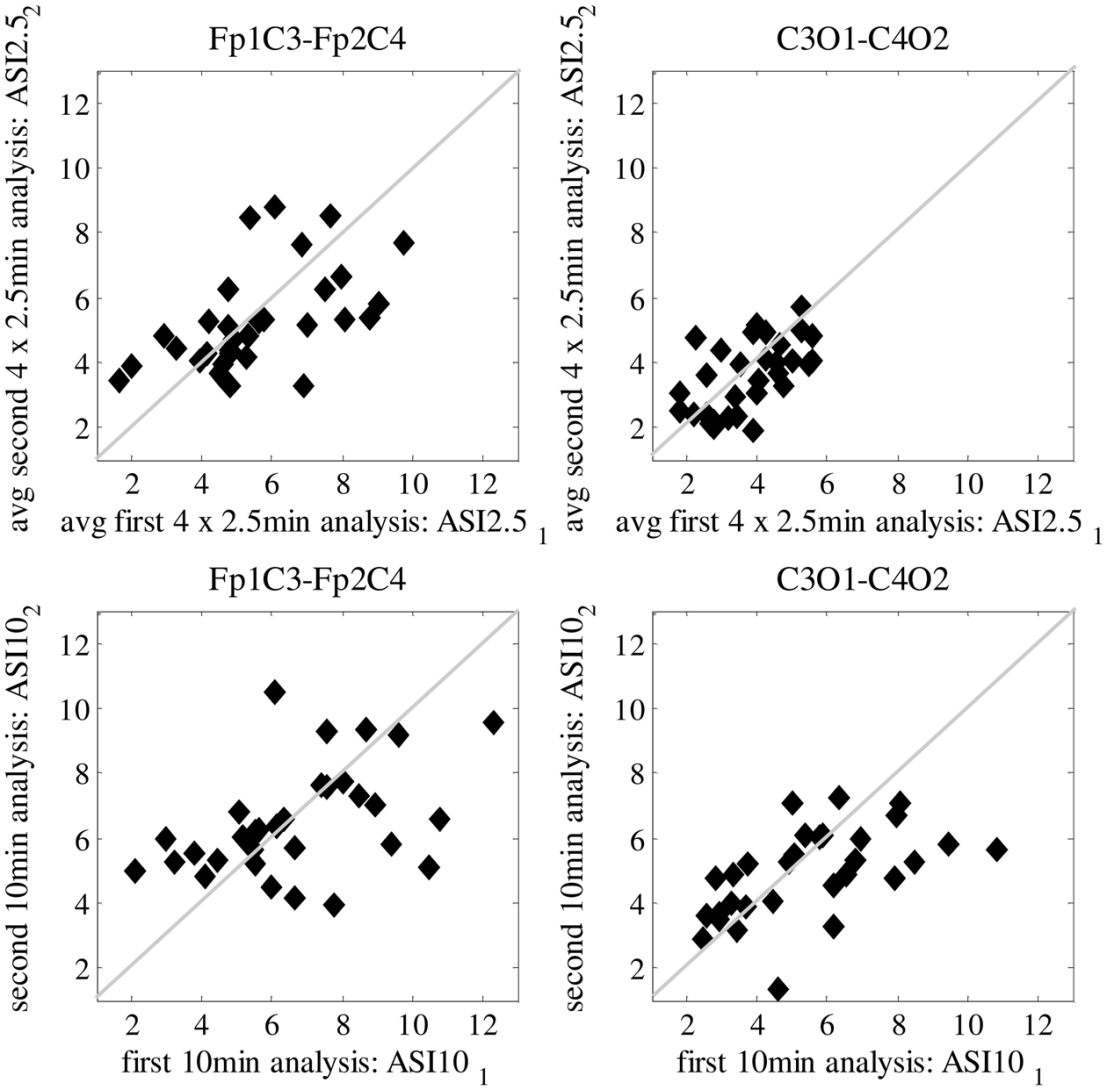
**Difference in mean-squared difference (MSD) between two analyzed periods of 10-min EEG, every dot representing a different patient**. Results are shown for Frontal-Centro (Fp-C) and Centro-Occipital (C-O) derivations for different analyzing window lengths (2.5/10 min). Best result with lowest MSD is shown in the upper right plot, obtained for average ASI values over 4 × 2.5 min analysis on C-O derivations.

In Figure 2, we show the ASI comparison of 10-min epochs for two clinically relevant channel pair combinations (Fp_1_C_3_-Fp_2_C_4_ and C_3_O_1_-C_4_O_2_). Visual inspection of the scatter plots shows that least scatter is found for C_3_O_1_-C_4_O_2_ in combination with smaller ASI windows (2.5 min). Quantitation of the scatter with MSD is summarized in Table 1 for all channel pair combinations and ASI window lengths. Out of all channel pairs tested, C_3_O_1_-C_4_O_2_ yielded the lowest MSD values. Out of all combinations of ASI windows tested, the average over 4 windows of 2.5 min led to the lowest MSD.

**Table 1:** **Mean squared difference (MSD) for different channel pairs and different ASI analyzing window lengths shown as an average for all 31 patients**.

	Fp_1_C_3_-Fp_2_C_4_	C_3_O_1_-C_4_O_2_	Fp_1_O_1_-Fp_2_O_2_	Fp_1_T_3_-Fp_2_T_4_	T_3_O_1_-T_4_O_2_	Mean MSD_ch
ASI_10 min	4.84	3.58	6.05	4.76	5.22	4.89
ASI avg_4 × 2.5min	2.99	1.06	1.63	1.15	2.02	1.77
ASI avg_2 × 5 min	9.90	4.23	8.60	5.51	5.25	6.70
ASI avg_2 × 2.5 min	3.21	2.22	3.82	2.91	3.74	3.18
ASI_5 min	7.59	4.71	5.71	5.38	9.05	6.49
Mean_MSD ASIwindows	5.71	316	5.16	3.94	5.06	

*Grand averages of the MSD values over all channel pair combinations and window lengths are shown as well. Examples shown in Figure 2 are underlined in this table*.

**Figure 3:**
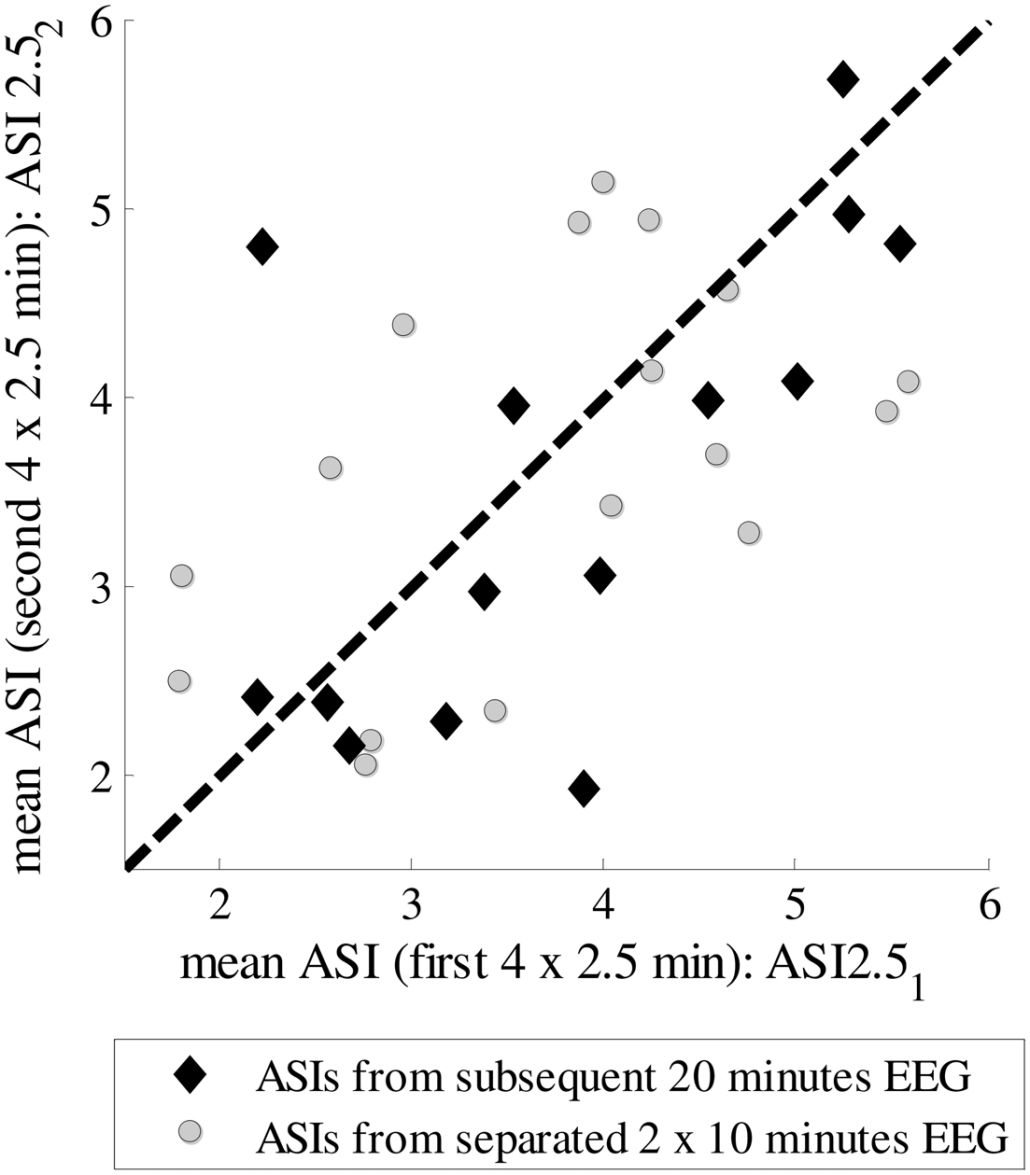
**ASIs for subsequent 10-min epochs from the same quiet sleep period or for separate 10-min epochs from two different quiet sleep periods**.

Next, we tested how stable the ASI value is for different selections of epochs in the whole EEG recording. We could find uninterrupted 20-min epochs of “artifact-free” quiet sleep in only a few patients, while in most of them the EEG was interrupted by arousals, movements or care procedures. Therefore, a scatter plot is shown for the analysis of an epoch of the same quiet sleep period (20 min) and of two epochs of different quiet sleep periods (2 × 10 min) (Figure 3). There is no significant difference in the MSDs between the epochs taken from the same vs. different quiet sleep epochs (mean MSD_subsequent = 1.061 and mean MSD_separated = 1.066, respectively; *p* = 0.12; Mann–Whittney *U*-test).

### Classification

**Figure 4:**
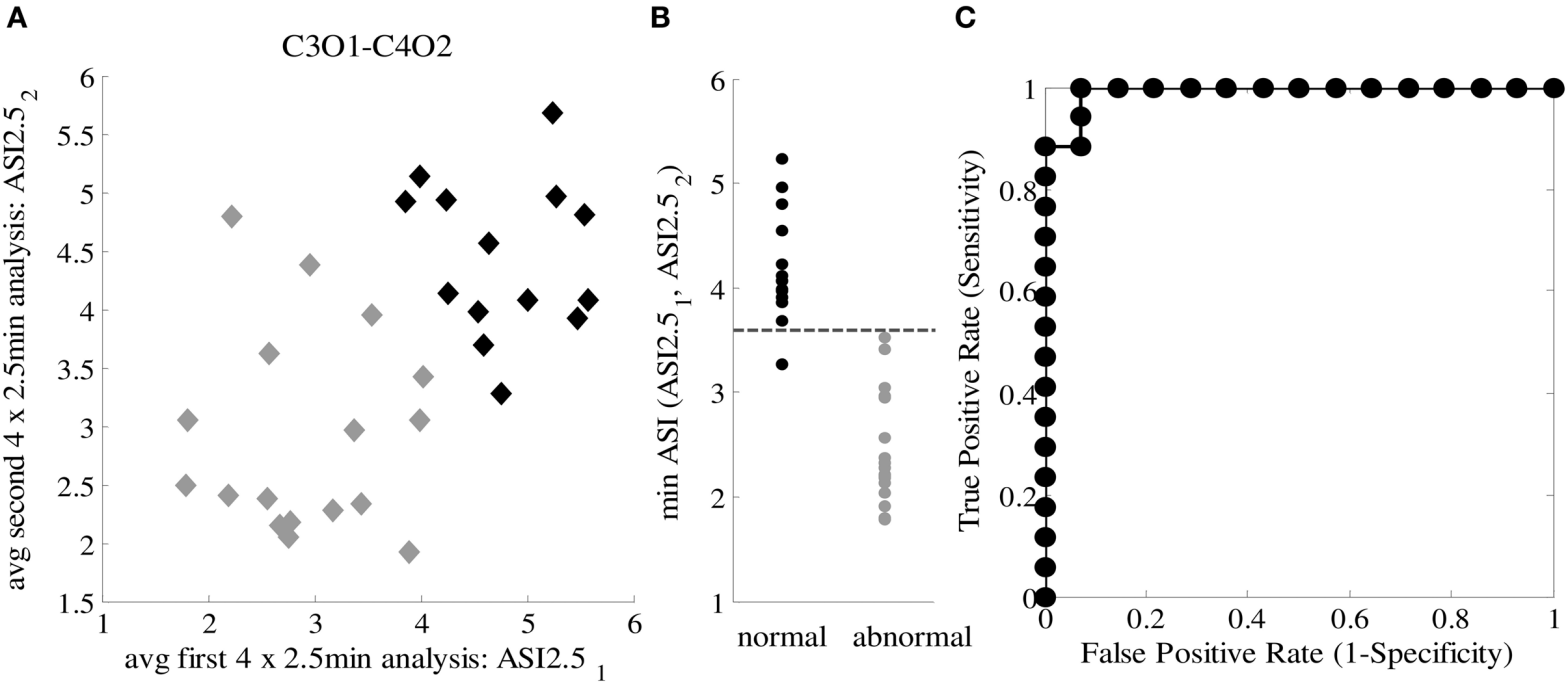
**(A)** ASI2.5_1_ and ASI2.5_2_ for both 10-min EEG segments from 31 patients for C-O derivation with the lowest MSD value. Two groups are specified: normal patients (black labels) and abnormal patients (gray labels). **(B)** Discrimination between normal and abnormal ASI taking the minimum ASI of ASI2.5_1_ and ASI2.5_2_ and thresholding (Th_ASI = 3.6). **(C)** ROC curve for classification, AUC = 0.971.

Using the two epochs of 10 min, we plotted the ASI values using the best channel pair (C3O1-C4O2) and window lengths (4 × 2.5 min), and labeled them according to clinical EEG judgments. As shown in Figure 4, the distributions of ASI values in the two groups are distinct. Comparison of the smaller of the two ASI values in each baby shows that the difference is highly significant (*p* < 0.001, *t*-test). A tentative cut-off set at ASI = 3.6 gives only one misclassified normal baby (pt #28) having an ASI below that limit, yielding a very high classification accuracy (96.77%). This patient was clinically classified as normal due to age-specific transients interpreted as “borderline” for the PMA age of 37.6 weeks (i.e., “immaturity”), which as a clinical finding does not need to be associated with globally abnormal function detected by ASI.

**Figure 5:**
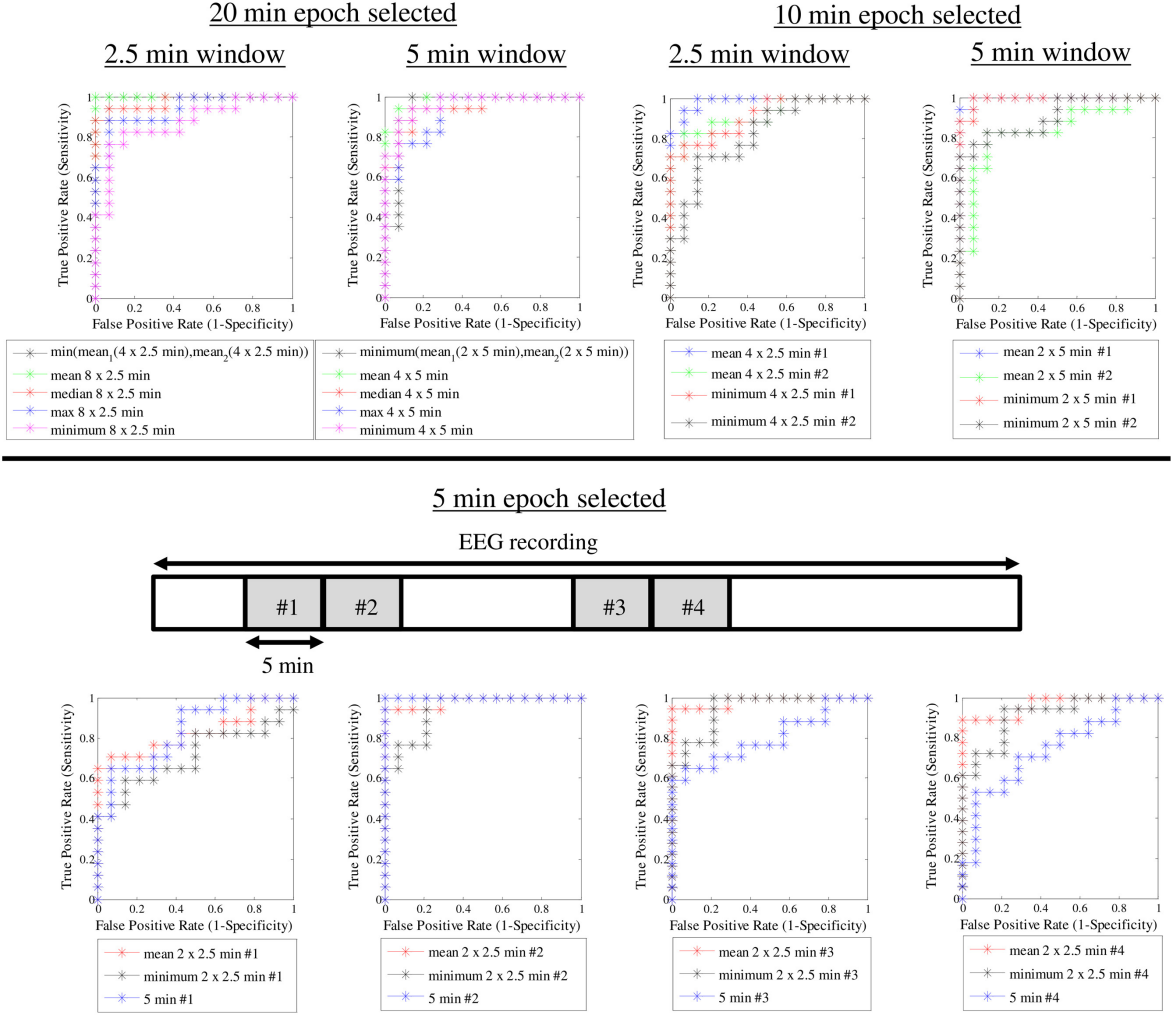
**ROC curves for different ASI window lengths and different epoch lengths**.

After observing such high classification accuracy using 2 × 10 min EEG epochs, we finally wanted to do a *post-hoc* analysis to see how the classification accuracy is affected by reduction in EEG data. This is important for clinical studies where the ideal 20 min of good quality EEG from quiet sleep is usually not available. Hence, we systematically examined a range of EEG lengths (5, 10 or 20 min), ASI window lengths (2.5, 5, and 10 min), as well as the way of combining the ASI values from multiple windows. Instead of attempting formal statistical comparisons, we aimed to provide useful practical answers by visually analyzing how the ROC curves change when the underlying data and analysis settings are changed. As an expected finding in the visual comparison of ROC curves, using more EEG data led to a generally better overall classification (Figure 5). We also observed that the length of the ASI window made a difference such that shorter ASI windows tended to yield higher classification accuracy. Moreover, the way of combining ASI estimates of multiple windows (mean vs. minimum vs. maximum) also appeared to affect the classification accuracy. Taking the mean ASI value from several windows seemed to produce more accurate classifications. However, qualitative comparison of ROC curves based on subsequent 5-min epochs showed that the classification accuracy varies considerably.

### Age dependence of ASI

**Figure 6:**
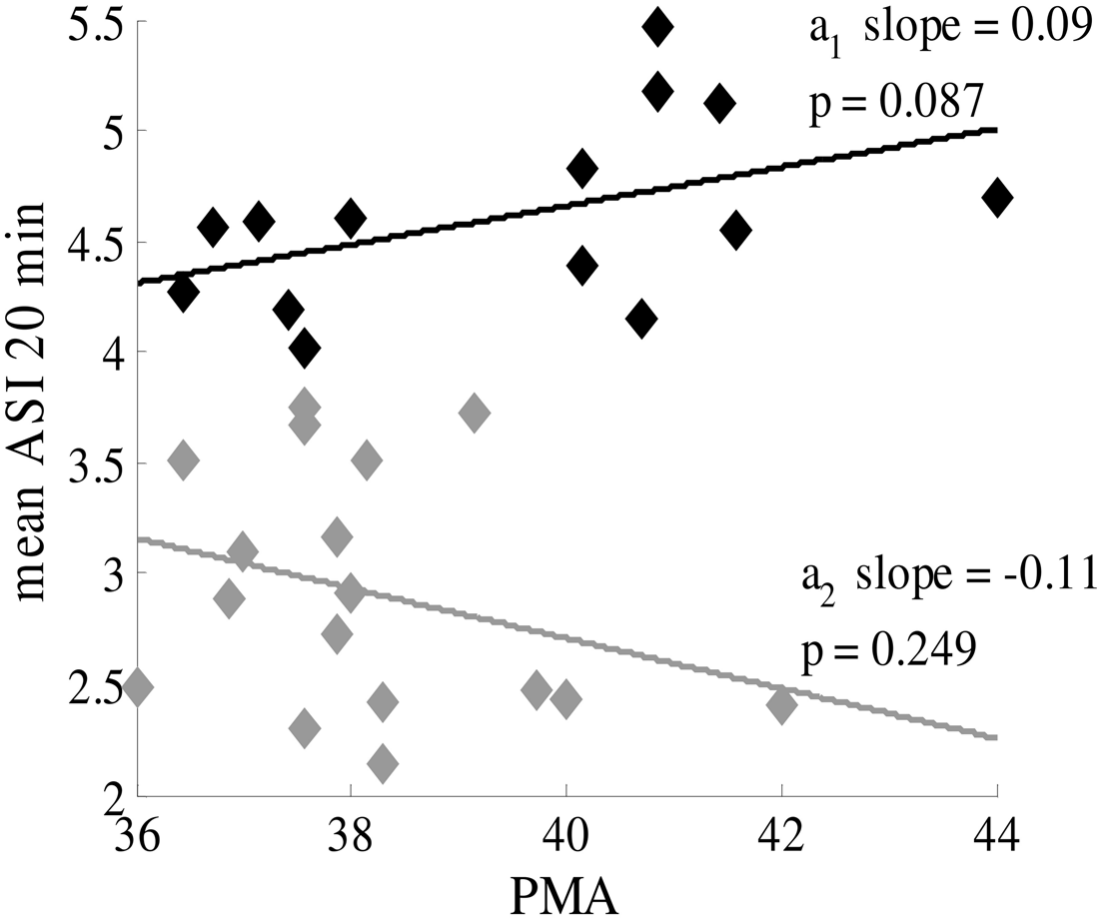
**ASI value is independent of postmenstrual age of the term infant (PMA >36 weeks)**. Black labels represent healthy patients with synchronous patterns; gray labels are patients with asynchronous patterns. The mean ASI value is calculated for manually-selected 20-min periods (8 × 2.5 min).

ASI was not found to significantly correlate with PMA in infants near term age (PMA >36 weeks) (Figure 6), and the slopes of the linear regression lines are very small. Hence, the same cut-off can be applied to distinguish normal and abnormal EEG throughout this age range, and no correction for PMA is needed.

## Discussion

Our study shows that IHS is a very robust feature of normal neonatal EEG, and that it can be readily quantified by using the novel metric ASI. We further show that ASI is able to reach the same classification that has been traditionally reached by a skilled EEG clinician when combining multiple visually-identified signal features. To the best of our knowledge, this study provides the first quantitative evidence in support of the old clinical thinking that disturbance of neonatal brain function is readily reflected in the interaction between hemispheres. We extend the earlier work with ASI by providing detailed assessment of how ASI performance is affected by variations in data length or analysis settings. As a conclusion, the results provide guidance for future employment of ASI in clinical work and research as well as in the development of automated EEG classifiers.

### Intraindividual stability and measurement stability: selection of small epochs

We found that the most stable ASI value was obtained by using the mean of multiple short-term ASI values, each estimated from a 2.5-min epoch. Physiologically, this observation suggests that long-range temporal correlations in the IHS are limited over time. Temporal correlations in amplitude fluctuations over several minutes have been shown in the adult brain (Linkenkaer-Hansen et al., 2001). Our recent observations in neonatal EEG datasets suggest, however, that only limited temporal correlations can be seen in the neonatal EEG signals (Matic et al., submitted), indirectly supporting the idea that ASI analyses may also best operate when using limited window lengths. Clinically and methodologically, using short segments offers several advantages in future studies and for possible implementation in brain monitoring algorithms. It is relatively easy to find several 2.5-min long undisturbed and sufficiently high quality (cf. Räsänen et al., 2013) EEG segments from neonatal recordings, while requesting longer (e.g., 10 min) continuous and undisturbed EEG epochs would pose a serious limitation in the future use of ASI. We suggest that the minimum duration of the recordings should be long enough to improve the statistical value and to overcome technical artifacts and subtle fluctuations in brain state at the multi-minute level. To this end, one should aim to collect a total of up to 20 min of quiet sleep data, which may come from successive sleep cycles. Reduction of the total data to 10 min will compromise individual diagnostic accuracy; however, it would only moderately decrease the utility of ASI at group level analyses (e.g., as an early biomarker).

### Channel pair combination and cut-off value

We found that the best intraindividual stability is obtained from using central-occipital derivation. Despite decades of clinical visual assessment of IHS, there are no established practices as to which derivations should be used. A common clinical experience is that temporal co-incidence of activity bursts varies between brain areas, especially when the IHS is decreased in sick babies. It is also common to have disturbing movement and muscle artifacts in the frontal and temporal channels. Our finding suggests that ASI is optimally measured from a derivation that, by co-incidence, also happens to be usually clean of artifacts. This helps implementation of ASI in future studies. Physiologically, this finding is compatible with current knowledge of neonatal brain networks. It is well established that anatomical neonatal networks grow first in post-central regions (Kostovic and Judas, 2010), and that neonatal EEG activity mostly occurs in posterior regions (André et al., 2010). It was also shown recently that there is a prominent posterior-parietal network in the newborn brain created by long-range amplitude correlations (Omidvarnia et al., 2014), which as a coupling mechanism comes close to what is measured by ASI. Taking these together, our observations suggest that the post-central networks may be the key driver of interhemispheric connectivity, and the traditionally observed loss of IHS in sick babies is probably due to changes in these posterior networks. Further studies are warranted to study whether different structural pathologies could give rise to altered IHS in a spatially selective manner.

### Age independency of ASI

We found a clear cut-off across the whole age range of babies, which suggests that future ASI implementation can disregard PMA when studying babies near term age. The younger infants in our group (PMA 36–38 weeks) showed more interindividual variability, which may reflect the as yet poorly established brain networks at that age. It has been shown that both the anatomical growth of callosal connections (Kostovic and Judas, 2010) and the appearance of visually observed IHS (Tharp et al., 1989; André et al., 2010) take place only a few weeks earlier, up to the 35th week of gestation. It is commonly seen in the clinic in these younger babies (data not shown) that the onset of quiet sleep may be markedly asynchronous and that synchrony increases within the quiet sleep period. Such dynamics could arise from functional instability in the young networks, which would readily explain a larger variability both within and between individuals.

### Future directions

Finding a high correlation between ASI and the traditional clinical visual EEG classification suggests that ASI may provide an objective and quantified global biomarker for neonatal brain function. Its design makes it physiologically reasoned; our current work makes it clinically benchmarked; and its technical properties make it transparent and straightforward to implement. Hence, it holds promise for becoming a useful feature in future clinical work and research, as well as in the construction of automated classifiers of neonatal EEG. In this context, finding quiet sleep periods in an automated way would be beneficial.

This research has potential value, as prematurity-associated injury of the subplate, early extrauterine environmental stimulation, and acquired brain lesions may induce a structural adaptation of synapses and, consequently, alter the normal differentiation of cortical connectivity or even disruption of the cortical network development. These altered brain functions, expressed as levels of inter- and intrahemisperic synchrony, may reflect transiently or permanently disturbed connectivity of the cortico-cortical and cortico-basal ganglia connections, even in the absence of standard neuroimaging abnormalities. Further work for clinical research may include ASI as an outcome parameter in standardized neurodevelopment follow-up data and may also include quantitative analyses of synchrony in preterm and term infants, to identify normative and altered patterns of development.

### Conflict of interest statement

The authors declare that the research was conducted in the absence of any commercial or financial relationships that could be construed as a potential conflict of interest.
