# Supplementary material for: Interhemispheric synchrony in the neonatal EEG revisited: activation synchrony index as a promising classifier
Source: Front Hum Neurosci. 2014 Dec 23;8:1030. doi: 10.3389/fnhum.2014.01030 (PMC4274973; doi:10.3389/fnhum.2014.01030)
Supplement: Supplementary file 1 [file DataSheet1.DOCX]

***Supplementary Materials***

**Interhemispheric synchrony in the neonatal EEG revisited: Activation Synchrony Index as a promising classifier**

**Ninah Koolen^1,2*†^, Anneleen Dereymaeker^3†^, Okko Räsänen^4^, Katrien Jansen^3^, Jan Vervisch^3^, Vladimir Matic^1,2^, Maarten De Vos^5,6^, Sabine Van Huffel^1,2^, Gunnar Naulaers^3^, Sampsa Vanhatalo^7^**

^1^Division STADIUS, Department of Electrical Engineering (ESAT), University of Leuven, Leuven, Belgium

^2^iMinds-KU Leuven Medical IT Department, Leuven, Belgium

^3^Department of Development and Regeneration, University of Leuven, Leuven, Belgium

^4^Department of Signal Processing and Acoustics, Aalto University, Espoo, Finland

^5^Department of Psychology, University of Oldenburg, Oldenburg, Germany

^6^Institute of Biomedical Engineering, Department of Engineering Science, University of Oxford, Oxford, UK

^7^Department of Children’s Clinical Neurophysiology, HUS Medical Imaging Center and Children’s Hospital, Helsinki University Central Hospital and University of Helsinki, Helsinki, Finland

*** Correspondence:** Ninah Koolen, Division STADIUS-BIOMED, Department of Electrical Engineering, University of Leuven, Kasteelpark Arenberg 10 - bus 2446, 3000 Leuven, Belgium.

[ninah.koolen@esat.kuleuven.be](mailto:ninah.koolen@esat.kuleuven.be)

^†^ These authors are joint first authors.

1. **Supplementary Table**

Supplementary Table 1: Detailed overview of the selected patients. With the following specifications:

PMA = postmenstrual age

GLOBAL ASSESSMENT:

1= ABNORMAL : at least 2 abnormal features

0= NORMAL: no more than 1 abnormal feature

FEATURE: continuity, symmetry, synchrony, voltage, sleep, variability, dysmaturity, normal age transients, abnormal patterns

NORMAL CONTINUITY = continuous EEG in AS and AW, TA > 38 weeks, 36-38 weeks TA or DC with IBI <10 sec,

SLEEP STAGE = sleep stage: QS (Quiet Sleep), AS (Active Sleep), AW (Active Wake), QS: DC (discontinuous pattern) or TA (tracé alternant)

AGE SPECIFIC TRANSIENTS: presence of different sleep stages: AS I, AS II en QS, specific transient: frontal ant dysrythmia, frontal sharps, occipital delta brushes, QS/AS

|  | **PMA** | **FEATURE: continuity, symmetry, synchrony, voltage, sleep, variability, dysmaturity, normal age transients, abnormal patterns** | | **GLOBAL ASSESSMENT** |
| --- | --- | --- | --- | --- |
| **PT 1** | 37 6/7 | continuity, synchrony, abnormal patterns | discontinuity in QS, no TA, a clearly percentage of EEG bursts occur asynchronously (greater than 1.5 seconds between onset of activity in each hemisphere) during DC EEG, excessive sharp wave transients | 1 |
| **PT 2** | 37 4/7 | continuity, synchrony, sleep, abnormal patterns | discontinuity in QS, no TA , a clearly percentage of EEG bursts occur asynchronously (greater than 1.5 seconds between onset of activity in each hemisphere) during DC EEG, preponderance of QS, excessive sharp wave transients | 1 |
| **PT 3** | 37 4/7 | continuity, synchrony, dysmaturity | discontinuity in Q,S no TA, a clearly excessive percentage of EEG bursts occur asynchronously (greater than 1.5 seconds between onset of activity in each hemisphere) during DC EEG, EEG that would be normal for an infant at least two weeks younger than the PMA, abundance of temporal theta | 1 |
| **PT 4** | 37 | continuity, synchrony, voltage, sleep, age transients, abnormal patterns | excessive discontinuity in all states, a clearly percentage of EEG bursts occur asynchronously (greater than 1.5 seconds between onset of activity in each hemisphere) during DC EEG, global voltage depression, lack of normal SWC, lack of normal transients, excessive sharp wave transients | 1 |
| **PT 5** | 36 6/7 | continuity, synchrony, variability, age transients, abnormal patterns | excessive discontinuity in all states, a clearly percentage of EEG bursts occur asynchronously (greater than 1.5 seconds between onset of activity in each hemisphere) during DC EEG, variability but no SWC , excessive sharp wave transients | 1 |
| **PT 6** | 38 | continuity, synchrony, variability, age transients, abnormal patterns | excessive discontinuity in all states, a clearly percentage of EEG bursts occur asynchronously (greater than 1.5 seconds between onset of activity in each hemisphere) during DC EEG, variability but no SWC , excessive sharp wave transients | 1 |
| **PT 7** | 40 1/7 | continuity, synchrony, sleep, age transients | normal continuity, normal synchrony, normal SWC, normal transients for age | 0 |
| **PT 8** | 36 | continuity, synchrony, dysmaturity | excessive discontinuity in QS, a clearly excessive percentage of EEG bursts occur asynchronously (greater than 1.5 seconds between onset of activity in each hemisphere) during DC EEG, EEG that would be normal for an infant at least two weeks younger than the PMA | 1 |
| **PT 9** | 37 4/7 | continuity, synchrony, abnormal patterns | discontinuity in QS, no TA, a clearly percentage of EEG bursts occur asynchronously (greater than 1.5 seconds between onset of activity in each hemisphere) during DC EEG, excessive sharp wave transients | 1 |
| **PT 10** | 44 | continuity, synchrony, sleep, age transients | normal continuity, normal synchrony, normal SWC, normal transients for age, some sharp wave transients | 0 |
| **PT 11** | 40 6/7 | continuity, synchrony, variability, age transients, abnormal patterns | excessive discontinuity in all states, a clearly percentage of EEG bursts occur asynchronously (greater than 1.5 seconds between onset of activity in each hemisphere) during DC EEG, variability but no SWC , excessive sharp wave transients | 1 |
| **PT 12** | 40 6/7 | continuity, synchrony, sleep, age transients | normal continuity, normal synchrony, normal SWC, normal transients for age | 0 |
| **PT 13** | 36 5/7 | continuity, synchrony, age transients | trace discontinue/TA in QS, normal synchrony, normal transients | 0 |
| **PT 14** | 38 | continuity, synchrony, sleep, age transients | normal continuity, normal synchrony, normal SWC, borderline transients for age: proportion of delta brushes in all states (rater 1: 1, rater 2:0) | 0 |
| **PT 15** | 37 1/7 | continuity, synchrony, sleep, age transients | normal continuity, normal synchrony, normal SWC, normal transients for age | 0 |
| **PT 16** | 38 2/7 | continuity, synchrony, dysmaturity | trace discontinue in QS, a clearly excessive percentage of EEG bursts occur asynchronously (greater than 1.5 seconds between onset of activity in each hemisphere) during DC EEG, EEG that would be normal for an infant at least two weeks younger than the PMA | 1 |
| **PT 17** | 42 | continuity, synchrony, sleep, age transients, abnormal patterns | trace discontinue in QS, a clearly percentage of EEG bursts occur asynchronously (greater than 1.5 seconds between onset of activity in each hemisphere) during DC EEG, global voltage depression, lack of normal transients, excessive occipital delta | 1 |
| **PT 18** | 37 1/7 | continuity, synchrony, age transients | trace discontinue/TA in QS, normal synchrony, normal transients | 0 |
| **PT 19** | 40 5/7 | continuity, synchrony, sleep, age transients | normal continuity, normal synchrony, normal SWC, normal transients for age | 0 |
| **PT 20** | 40 1/7 | continuity, synchrony, sleep, age transients | normal continuity, normal synchrony, normal SWC, normal transients for age | 0 |
| **PT 21** | 38 2/7 | continuity, synchrony, symmetry, abnormal patterns | trace discontinue in QS, no TA, a clearly percentage of EEG bursts occur asynchronously (greater than 1.5 seconds between onset of activity in each hemisphere) during DC EEG, 2:1 difference in voltages and a clear disparity of background features between homologous regions of the two hemispheres, excessive sharp transients | 1 |
| **PT 22** | 36 3/7 | continuity, synchrony, dysmaturity | trace discontinue in QS, a clearly excessive percentage of EEG bursts occur asynchronously (greater than 1.5 seconds between onset of activity in each hemisphere) during DC EEG, EEG that would be normal for an infant at least two weeks younger than the PMA | 1 |
| **PT 23** | 38 1/7 | continuity, synchrony, sleep, abnormal patterns | discontinuity in all states, a clearly percentage of EEG bursts occur asynchronously (greater than 1.5 seconds between onset of activity in each hemisphere) during DC EEG, lack of normal SWC, excessive sharp wave transients | 1 |
| **PT 24** | 41 3/7 | continuity, synchrony, sleep, age transients | normal continuity, normal synchrony, normal SWC, borderline sharp wave transients | 0 |
| **PT 25** | 39 5/7 | continuity, synchrony, dysmaturity | trace discontinue in QS, a clearly excessive percentage of EEG bursts occur asynchronously (greater than 1.5 seconds between onset of activity in each hemisphere) during DC EEG, EEG that would be normal for an infant at least two weeks younger than the PMA | 1 |
| **PT 26** | 37 6/7 | continuity, synchrony, symmetry, variability, age transients, abnormal patterns | trace discontinue in all states, a clearly percentage of EEG bursts occur asynchronously (greater than 1.5 seconds between onset of activity in each hemisphere) during DC EEG, 2:1 difference in voltages and a clear disparity of background features between homologous regions of the two hemispheres, excessive sharp transients | 1 |
| **PT 27** | 40 6/7 | continuity, synchrony, sleep, age transients | normal continuity, normal synchrony, normal SWC, normal transients for age | 0 |
| **PT 28** | 37 4/7 | continuity, synchrony, dysmaturity | trace discontinue/TA in QS, normal synchrony, borderline dysmaturity: EEG that would be normal for an infant at least 1 week younger than the PMA | 0 |
| **PT 29** | 36 3/7 | continuity, synchrony, sleep, age transients | normal continuity, normal synchrony, normal SWC, normal transients for age | 0 |
| **PT 30** | 41 6/7 | continuity, synchrony, sleep, age transients | normal continuity, normal synchrony, normal SWC, normal transients for age | 0 |
| **PT 31** | 39 1/7 | continuity, synchrony, dysmaturity, abnormal patterns | trace discontinue in QS, a clearly excessive percentage of EEG bursts occur asynchronously (greater than 1.5 seconds between onset of activity in each hemisphere) during DC EEG, EEG that would be normal for an infant at least two weeks younger than the PMA, borderline sharp wave transients (rater 1: 1, rater 2:0) | 1 |

1. **Technical details ASI algorithm**

During pre-processing, the two analyzed input channels are first downsampled to 50 Hz and signal content at higher frequencies is emphasized using a first order finite impulse response (FIR) filter of the form H(z) = 1-0.95z^-1^. Smoothed signal amplitude envelopes are computed from the pre-processed signals by taking the Fast Fourier Transform (FFT) using a Hamming window length of 2 seconds and a window step size of 100 ms and by summing over the resulting amplitude spectrum in the 1.5–20 Hz range. Notably, this frequency range deviates slightly from the range of 1.5-25Hz reported in the original work, but based on the optimization results in our original work (see Fig 2 in Räsänen et al., 2013) this will not significantly affect the results.

In order to statistically estimate cross-channel dependencies in the discrete probability domain, the signal envelopes are quantized into eight discrete amplitude levels (Räsänen et al., 2013). Quantization is performed by estimating quantization levels using a standard k-means clustering algorithm and then assigning each envelope sample to the nearest cluster (indexed from 1 to 8). As a result, both channels *A* and *B* are described as discrete sequences *X_A_ = {a_1_, a _2_, …, a_N_}* and *X_B_ = {b_1_, b_2_, …, b_N_}* with subscripts denoting the time index, each *a_t_,b_t_ ∈ {1, 2, …, 8}*, and one element occurring every 100 ms (the FFT step size). Then the so-called energy weighted temporal dependency function (EDTF) is computed between the two quantized envelopes (Räsänen et al., 2013) (formula 1). EDTF is a mutual information-based measure that describes the level of deviation from a statistical independence assumption between the two analyzed signals. However, in contrast to mutual information, it also takes into account the amount of signal energy associated with the observed dependencies. When computed for a number of different time lags between the two channels (default: τ = [-5, 5] s with a 100 ms step length), i.e., by delaying first of the channels with respect to the second one by τ seconds, the resulting ETDF curve characterizes the amount of signal synchrony between the two channels at each of the analyzed lags.

| $ETDF\left( \tau\right)=\sum_{a,b} AMP\left( a \right)AMP(b)\frac{p_{\tau}{(a,b)}^{2}}{p\left( a \right)p(b)}$ | (1) |
| --- | --- |

In the equation, *a* and *b* denote the quantization levels in the two channels respectively. *p_τ_ (a,b)* is the probability of observing level *a* in first channel and level *b* in second channel when the second channel is delayed by *τ* units with respect to the first one, *p(a)* is the probability of observing level *a* in the first channel and *p(b)* for level *b* in the second channel. *AMP(a)* and *AMP(b)* are the mean amplitudes corresponding to quantization levels *a* and *b*. All probabilities are computed by simply counting the relative frequencies of lagged state pairs {*a,b*} and individual states across the entire analysis time window.

In a next step, the ETDF curve is normalized to have zero mean (formula 2). The final Activation Synchrony Index (ASI) value can be derived from the normalized ETDF curve as the ratio of the ETDF value at zero lag (τ = 0) over the expected (mean) value of the ETDF across the analyzed 10 s range [-5, 5] s (formula 3).

| ${EDTF}_{n}\left( \tau\right)=EDTF\left( \tau\right)-\min\left( EDTF\left( x \right),x \epsilon\left[ -5, 5 s \right] \right)$  $ASI=\frac{{EDTF}_{n}(\tau=0)}{E\left\{ {ETDF}_{n}\left( \tau\right) \vert\tau\epsilon[-5, 5 s] \right\}}$ | (2)  (3) |
| --- | --- |
